# Co-Immobilization of RizA Variants with Acetate Kinase for the Production of Bioactive Arginyl Dipeptides

**DOI:** 10.3390/molecules27144352

**Published:** 2022-07-07

**Authors:** Sven Bordewick, Ralf G. Berger, Franziska Ersoy

**Affiliations:** Institut für Lebensmittelchemie, Gottfried Wilhelm Leibniz Universität Hannover, Callinstraße 5, 30167 Hannover, Germany; rg.berger@lci.uni-hannover.de (R.G.B.); franziska.ersoy@lci.uni-hannover.de (F.E.)

**Keywords:** covalent immobilization, co-immobilization, biocatalysis, l-amino acid ligase, acetate kinase, ATP regeneration, arginyl dipeptides, salt-taste enhancer, protein engineering, antihypertensive dipeptide

## Abstract

The biocatalytic system comprised of RizA and acetate kinase (AckA) combines the specific synthesis of bioactive arginyl dipeptides with efficient ATP regeneration. Immobilization of this coupled enzyme system was performed and characterized in terms of activity, specificity and reusability of the immobilisates. Co-immobilization of RizA and AckA into a single immobilisate conferred no disadvantage in comparison to immobilization of only RizA, and a small addition of AckA (20:1) was sufficient for ATP regeneration. New variants of RizA were constructed by combining mutations to yield variants with increased biocatalytic activity and specificity. A selection of RizA variants were co-immobilized with AckA and used for the production of the salt-taste enhancers Arg-Ser and Arg-Ala and the antihypertensive Arg-Phe. The best variants yielded final dipeptide concentrations of 11.3 mM Arg-Ser (T81F_A158S) and 11.8 mM Arg-Phe (K83F_S156A), the latter of which represents a five-fold increase in comparison to the wild-type enzyme. T81F_A158S retained more than 50% activity for over 96 h and K83F_S156A for over 72 h. This study provides the first example of the successful co-immobilization of an l-amino acid ligase with an ATP-regenerating enzyme and paves the way towards a bioprocess for the production of bioactive dipeptides.

## 1. Introduction

l-amino acid ligases (LALs; EC 6.3.2.28) are a relatively novel enzyme class that synthesizes dipeptides from their respective amino acids. They belong to the ATP-grasp superfamily and hydrolyze ATP to ADP and P_i_ to catalyze the amide bond formation through an acyl phosphate intermediate [1]. The first LAL ywfE (also called BacD) was discovered in *B. subtilis* in 2005 [2]. Since then, LALs with different specificities have been identified [3,4,5]. The LAL RizA from *B. subtilis* NBRC3134 has a very high specificity for the synthesis of dipeptides containing an N-terminal arginine (Arg-X), many of which (e.g., Arg-Ser, Arg-Ala, Arg-Gly) have been found to have salt-taste enhancing effects [6,7,8,9]. Additionally, Arg-Phe is a potential antihypertensive [5,10]. Due to the very high cost of the cofactor ATP, we previously worked on employing acetate kinase (AckA) from *E. coli* to regenerate ATP from acetyl phosphate (AcP), which is cheaply accessible through acetylation of phosphoric acid by acetic anhydride [11,12]. The optimized enzyme system produced up to 23 mM Arg-Ser (46% yield) while only necessitating 0.5 mM ATP [13].

Immobilization can significantly improve the economic viability of a bioprocess by increasing stability, enabling reusability and thus decreasing production costs [14]. Additionally, it can also improve downstream processing through easier enzyme removal and improve enzyme properties like activity or specificity. Immobilization techniques are usually classified by the method through which a carrier is bound [14,15,16,17,18]. The strongest interaction is achieved through covalent immobilization. The induced rigidity can lead to high increases in stability [19,20], but also decreases in activity, as enzymatic mobility might be restricted or residues of the active site blocked [17]. Additionally, covalent methods often employ harsh reagents or reaction conditions that can potentially denature the protein [21]. One particularly mild covalent coupling method is the usage of carriers activated with esters of *N*-hydroxysuccinimide (NHS-esters). Both N-terminal amino groups and lysine residues form strong amide bonds after immobilization for one hour at near physiological pH [22,23]. Recently, NHS-activated agarose was used for food-grade immobilization of asparaginase for the removal of acrylamide [24]. Agarose beads have remarkable mechanical, chemical and biological stability, which can be further improved by cross-linking [25,26].

The co-immobilization of multiple enzymes is reminiscent of the organization of enzymes into enzyme complexes in living cells with short diffusion distances between enzymes in a reaction sequence [25,27]. It is also helpful if intermediate products are unstable or inhibitory and accumulation is not wanted [25]. Apart from enzyme cascades, cofactor regeneration is one of the most prominent applications. Regeneration of the electron donor NAD(P)H is a challenge for the application of many oxidoreductases. Examples include ketoreductases or xylose dehydrogenase, which were co-immobilized with glucose or alcohol dehydrogenase, respectively [28,29,30,31]. Regeneration of ATP has been described in the co-immobilization of glutathione synthetase with a polyphosphate kinase [32]. Perhaps the most impressive example is the co-immobilization of a thermostable acetate kinase with a pantothenate kinase in the multi-enzymatic cascade for the production of the potential antiviral islatravir [33]. This work also sets a good example of the possibilities of integrating immobilization and protein engineering: five of the nine enzymes were engineered, and three were (co-)immobilized. Protein engineering can act as the means to prepare an enzyme for successful immobilization (e.g., by improving its stability or introducing sites for immobilization like affinity tags) [28,34].

In a previous study, variants of RizA with improved activity and specificity for the production of several dipeptides were created [35]. After improving the applicability of RizA for a future industrial process both by establishing ATP regeneration [13] and protein engineering, co-immobilization was the next step towards this goal. Covalent immobilization using NHS-agarose was performed on both the unmodified enzymes and a selection of RizA variants generated through a combination of mutations from the previous study to examine how these mutations would affect immobilization. Lastly, the best variants for the production of the salt-taste enhancer Arg-Ser and the antihypertensive Arg-Phe were recycled for multiple reaction cycles to investigate the reusability of the immobilisates.

## 2. Results and Discussion

### 2.1. (Co-)Immobilization Conditions

**Figure 1:**
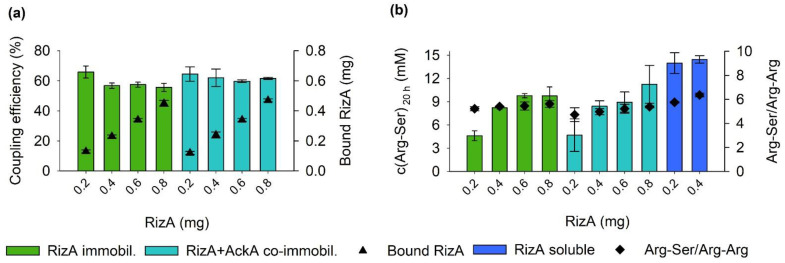
Immobilization of RizA in comparison to co-immobilization of RizA and AckA. (**a**) Coupling efficiencies and calculated amount of RizA bound to the agarose support. (**b**) Product and side product formation in 500 µL reaction volume with 30 mM Arg and Ser after 20 h at 25 °C. Reactions were performed in duplicates, with error bars representing the upper and lower value.

To examine whether co-immobilizing both RizA and AckA into a single immobilisate was viable, immobilizations were set up with different amounts of RizA ranging from 0.2 to 0.8 mg and 20 mg NHS-agarose. AckA was added in a mass ratio of 5:1 (RizA:AckA). For comparison, immobilisates containing only RizA were produced analogously. Protein concentrations before and after immobilization were measured, and the difference was used to calculate the coupling efficiency and determine the apparent amount of RizA immobilized on the agarose support (Figure 1a).

Coupling efficiencies were similar regardless of the applied amount of protein and ranged from 56 to 66%. Thus, the amount of RizA bound to the support increased approximately linearly with the applied amount. Overall, the coupling efficiencies were lower than anticipated as the manufacturer states that they are “typically greater than 80%, regardless of the ligand’s molecular weight or pI” [22]. However, the isoelectric point (pI) of RizA was calculated to be 5.9, and acidic pIs up to 5.9 were previously identified to inhibit coupling to agarose supports using NHS-esters [36]. Another possibility is that tricine, a secondary amine, in the purification/coupling buffer competed with the enzymes for coupling to the support, although both the lower basicity and steric hindrance of the amino nitrogen in tricine should generally limit this potential competition [37]. Lastly, there is competition between the desired aminolysis of the NHS-ester by the protein amino groups and hydrolysis by water, which can also reduce the coupling efficiency [38].

The immobilisates were used in a batch setup in reaction tubes for the biocatalytic production of Arg-Ser. The reactions with only immobilized RizA contained 0.05 mg soluble AckA. Control reactions with 0.2 and 0.4 mg soluble RizA and 0.05 mg AckA were included. After 20 h, concentrations of the product Arg-Ser and side product Arg-Arg were determined by RP-HPLC (Figure 1b). While the reactions with the lowest amount of RizA led to a decreased product concentration, all other immobilisates produced similar product concentrations of around 9 mM Arg-Ser. The reaction with 0.8 mg co-immobilized RizA produced 11 mM, but with a larger error bar. No substantial differences were found between immobilization of only RizA and the co-immobilization of both RizA and AckA. In comparison with the reactions with soluble RizA, the immobilisates showed a significantly lower activity since 0.2 mg (co-)immobilized RizA produced approximately 5 mM and 0.2 mg soluble RizA 14 mM Arg-Ser. Due to the incomplete coupling, part of the lower activity can be attributed to the lower actual amount of enzyme present in the reactions. However, higher enzyme amounts during the immobilization did not increase the product concentration. Decreases in activity are a known drawback of covalent immobilization techniques [14,15]. Apart from denaturing during the immobilization procedure, the rigid covalent attachment can distort the enzyme structure or bind it in such an orientation that the transfer of substrates and products to and from the enzyme is limited [17]. Side product formation was similar in all reactions with specificity ratios (Arg-Ser/Arg-Arg) of approximately 6.

**Figure 2:**
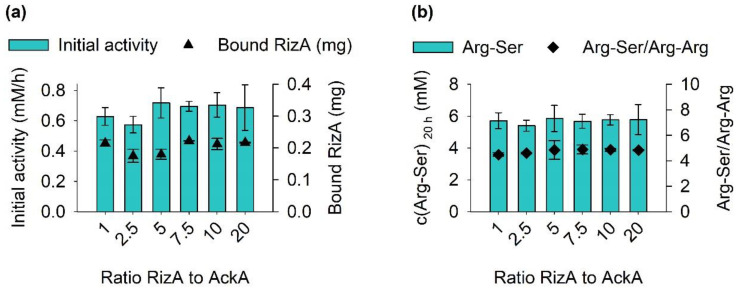
Effect of different ratios of RizA and AckA during co-immobilization. 0.4 mg of RizA were used, as well as a corresponding amount of AckA. Reaction conditions were identical to Figure 1. (**a**) Initial activities were determined in the first two hours of reaction time with linear regression. All R^2^ were above 0.96. (**b**) Final product and side product concentrations were determined after 20 h. Reactions were performed in duplicates with error bars representing the upper and lower value.

Next, the ratio between both enzymes was optimized. Former experiments with free RizA and AckA showed that supplying AckA in a ratio of 4 to 1 was sufficient to supply ATP for the reaction [13]. To examine this factor for the co-immobilized enzyme system, reactions were set up with a constant amount of 0.4 mg RizA and different mass ratios of AckA ranging from 1:1 to 20:1 (RizA:AckA). During the first two hours, samples were taken each half-hour to determine the initial activities (Figure 2a). The final product concentrations were measured after 20 h (Figure 2b).

The two highest additions of AckA decreased the initial activities of the immobilisates slightly, possibly indicating a negative effect of the bound AckA on RizA. Overall, no large differences in product or side product formation were determined, and even the smallest addition of AckA was sufficient to drive ATP regeneration at a low ATP concentration of 0.5 mM. For all following experiments, AckA was added in a ratio of 1 to 10. While the lowest addition of 1 to 20 would be the most efficient, a higher addition was chosen to not risk limiting the cofactor supply, since the demand for ATP regeneration is dependent on the reaction conditions.

### 2.2. Co-Immobilization of RizA Variants

#### 2.2.1. Combination of Mutations to Yield Improved Variants

In a previous study, a total of 21 variants of RizA were created and characterized [35]. Arg-Ser formation was increased up to 41% in T81F, while K83F/R, S84F and A158S also substantially increased it. Arg-Ala formation was increased by T81F, K83F and A158S, and also by S156A/F and D376E. The largest increase was found for S156A, which increased Arg-Phe formation by 270% in comparison to the wild type. Additionally, Arg-Phe formation was also increased by S156F and D376E.

**Figure 3:**
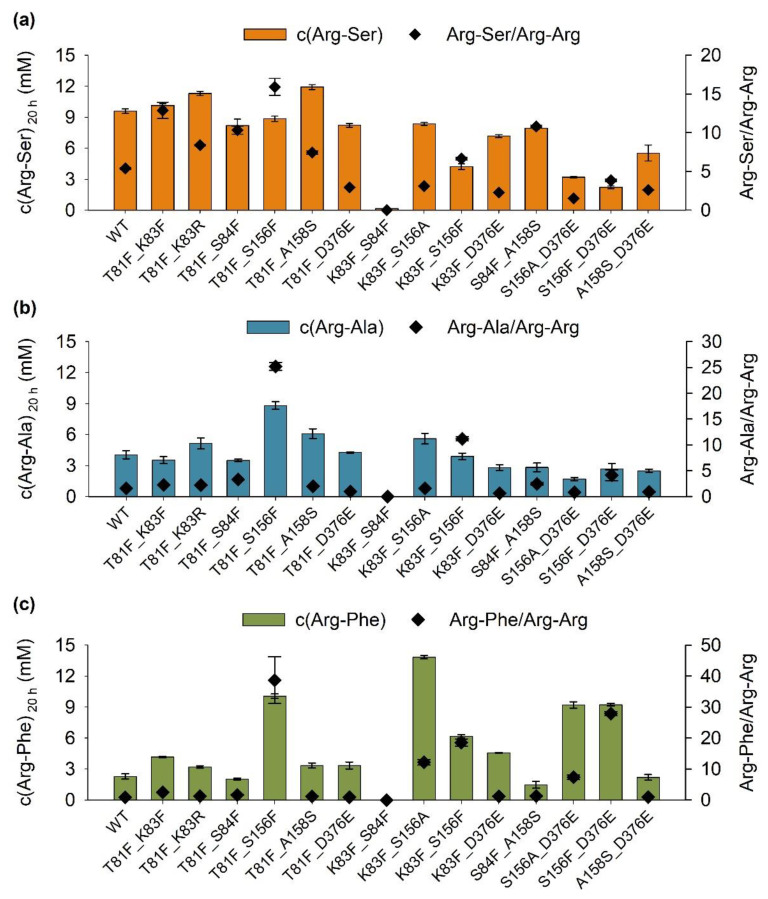
Production of Arg-X dipeptides by soluble variants containing two mutations. Reactions were set up in triplicates and contained 30 mM each Arg and (**a**) Ser, (**b**) Ala or (**c**) Phe. A total of 0.2 mg/mL RizA variant and 0.1 mg/mL AckA were used.

Here, these 8 best mutations from the previous study were combined to generate 14 new variants. For Arg-Ser, most combinations had no beneficial effect and showed lower product concentrations than the wild type (Figure 3a). Only T81F_K83R and T81F_A158S yielded higher Arg-Ser concentrations, with the last showing the highest with 12 mM. Variants also increased specificity up to a ratio of 13 for T81F_K83F. The highest specificity (15) was detected for T81F_S156F, but with a slightly reduced product formation.

For Arg-Ala, T81F_S156F also displayed the highest specificity ratio, in this case, 25, along with the highest product formation of 9 mM, which was more than a two-fold increase in comparison to the wild-type enzyme (Figure 3b). T81F_K83R, T81F_A158S and K83F_S156A also increased product formation, but to a lesser degree. Combinations with D376E showed no increase in comparison to the wild type. Similar to the other two products, T81F_S156F led to a large increase in specificity to 39 for the production of Arg-Phe, while also significantly increasing product formation to 10 mM (Figure 3c). Only K83F_S156A generated more product (14 mM). K83F_S156F, S156A_D376E and S156F_D376E also increased product formation and specificity.

Overall, most combinations of mutations had no additive effect, which is a known phenomenon and challenge in protein engineering [39]. The two significant exceptions were T81F_S156F, which increased the Arg-Ala concentration to 8.8 mM (best single variant D376E: 5.6 mM) and K83F_S156A, which increased Arg-Phe concentration to 13.8 mM (best single variant S156A: 12.1 mM). While T81F_A158S did not increase Arg-Ser formation over T81F, it raised the specificity ratio to 12 in comparison to 9. With the exception of D376E, all of the examined mutations were located in the binding pocket for the C-terminal substrate [35]. Interestingly, in most cases where combinations of mutations had a beneficial effect, the two residues were located on different loops (e.g., T81F and A158S). Since increasing the number of mutations can destabilize an enzyme [40], not accumulating too many changes in one part of the protein is likely a sound strategy. In contrast, mutation of both neighboring residues K83 and S84 to the large phenylalanine was the only example where activity was completely destroyed. While the combination of mutations did not lead to considerable increases in activity in most cases, it produced multiple variants with the highest specificities to date. While the highest specificity for a single variant was 12 (S84F for Arg-Ser), T81F_S156F displayed specificities of up to 39 for Arg-Phe production. It was the variant with the lowest side product formation and showed the highest specificity ratios for Arg-Ser and Arg-Ala production as well.

#### 2.2.2. Immobilization of RizA Variants

**Figure 4:**
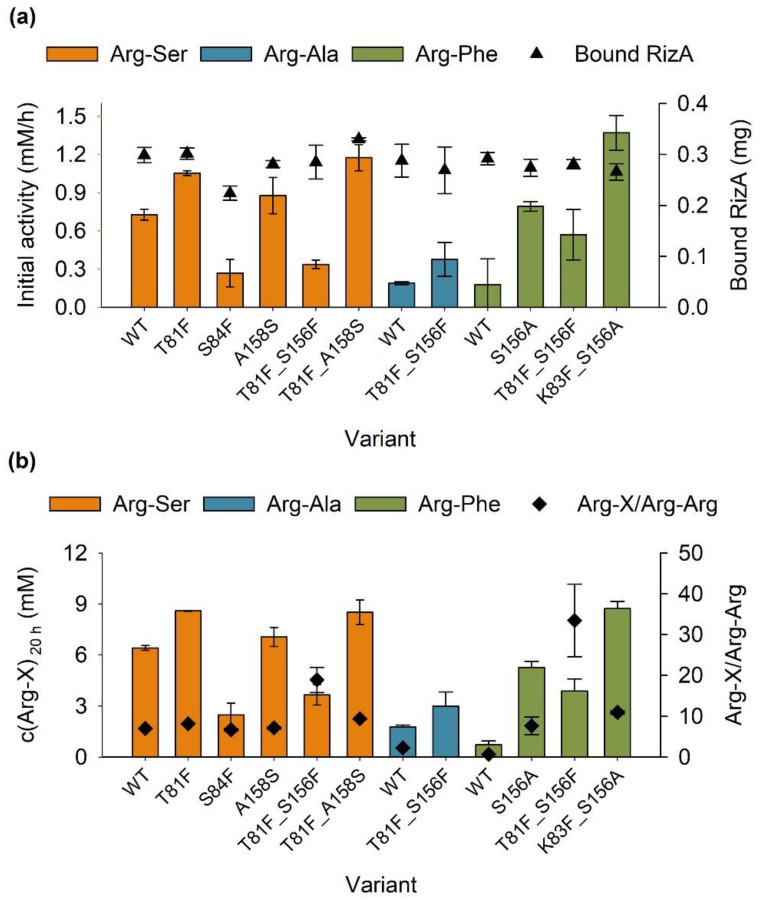
Co-immobilization of RizA variants. A total of 0.4 mg of RizA variant and 0.04 mg AckA were used. Reaction conditions were identical to Figure 1. (**a**) Initial activities were determined in the first two hours of reaction time with linear regression. All R^2^ were above 0.95. (**b**) Final product and side product concentrations were determined after 20 h. Reactions were performed in duplicates with error bars representing the upper and lower value.

A selection of nine RizA variants from both this and the previous study were chosen based on activities and specificities. They were co-immobilized analogously to the wild-type enzyme with AckA and characterized. Most variants were immobilized with similar efficiency to the wild type, corresponding to 0.27 to 0.33 mg RizA bound to the support from an application of 0.4 mg (Figure 4a). S84F could only be immobilized to a reduced degree (0.22 mg). Both the initial activities (Figure 4a) and the final product concentrations after 20 h (Figure 4b) were determined.

For Arg-Ser, both T81F and T81F_A158S showed the highest final product concentration, but T81F_A158S had higher initial activity and the highest specificity ratio of 9 compared to 8 for T81F and 7 for the wild type. S84F and T81F_S156F showed the lowest Arg-Ser formation. S84F was the variant with the second-highest product formation of Arg-Ser [35], but lost the majority of its activity due to immobilization. The unusually low binding to the support also contributed to the decrease in activity. For Arg-Ala, T81F_S156F had a similar increase in activity compared to the immobilized wild type and reached a slightly improved final product concentration of 3 mM, although with very high specificity as no Arg-Arg could be detected. Of the three dipeptides, the variants generated the strongest improvements for the production of Arg-Phe. In comparison to the wild type, the product concentration of 9 mM generated by K83F_S156A represents a nine-fold increase with a substantial increase in specificity from 1 to 11. The highest overall specificity of 33 was also generated for Arg-Phe by T81F_S156F and went along with an increased product concentration of 4 mM.

### 2.3. Characterization of the Immobilisates

**Figure 5:**
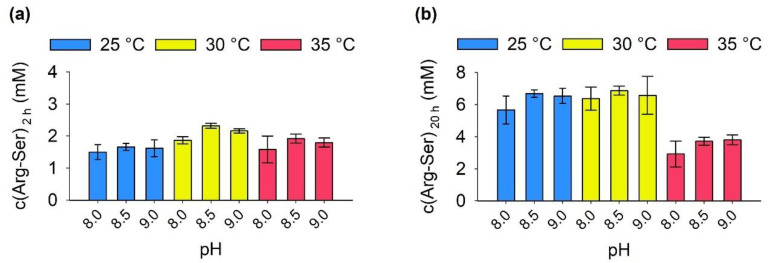
Effect of reaction temperature and pH. A total of 0.4 mg of RizA and 0.04 mg AckA were used for immobilization. Additionally, 50 mM each Arg and Ser were used. Product concentrations were measured (**a**) after 2 and (**b**) after 20 h reaction time.

In a first step, reactions were performed with wild-type immobilisate at different reaction temperatures and pH and characterized in terms of initial (Figure 5a) and final Arg-Ser product concentration (Figure 5b). All reaction conditions led to similar initial Arg-Ser concentrations between 1.5 and 2.3 mM after 2 h, with the reaction at 30 °C and pH 8.5 producing the highest concentration. However, after 20 h, all reactions at 25 and 30 °C yielded similar product concentrations between 6 and 7 mM, while reactions at 35 °C only contained approximately 4 mM. This was in line with the results for free RizA and AckA where 25 and 30 °C produced similar results, and 37 °C led to significantly lower product concentrations. Since 37 °C has been determined as the temperature optimum of free RizA [6], this disparity was likely related to the cofactor regeneration. Since the initial product formation at 35 °C was similar, the limitation manifested at longer reaction times. AckA was determined to be stable up to 40 °C [41]. Hydrolysis of both ATP and AcP is known to accelerate at higher temperatures [42]. While approximately 20% of the latter is hydrolyzed after 5 h at 20 °C, it is hydrolyzed completely in 3 to 5 h at 60 °C [43].

For the free enzymes, increasing the amino acid concentrations substantially improved product formation [13]. Here, the increase in the substrate to 50 mM while maintaining the applied amount of RizA at 0.4 mg did not increase product formation in the 25 °C and pH 8.5 reaction compared to the previous reactions at 30 mM under comparable reaction conditions (Figure 2b). In an attempt to increase the product concentration, the applied amount of RizA was increased to 0.8 mg for all following experiments.

**Figure 6:**
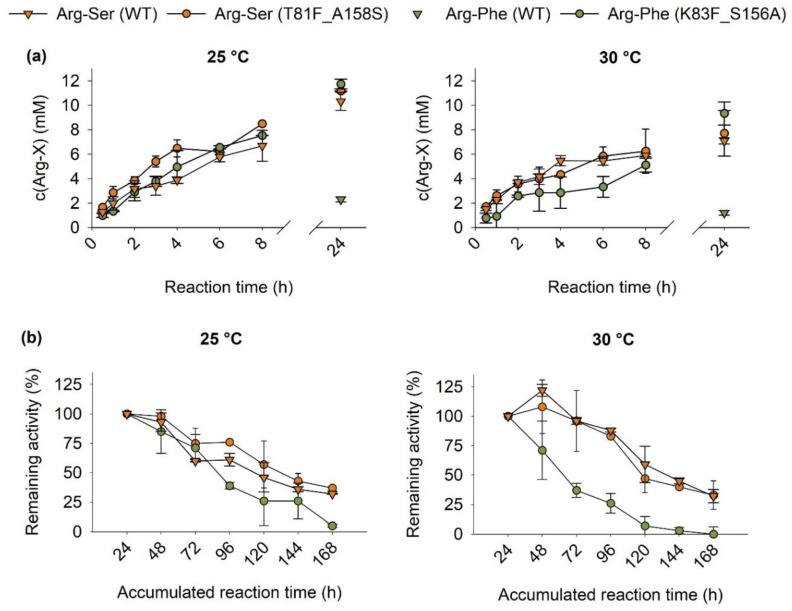
Effect of reaction temperature on (**a**) the time course of dipeptide formation and (**b**) reusability of the immobilisates of RizA wild type and variants. The remaining activity was calculated as the product concentration reached after an additional cycle of 24 h in comparison to that after the first cycle. A total of 0.8 mg RizA and 0.08 mg AckA were used for immobilization. Additionally, 50 mM Arg and Ser were used. No time course and remaining activity could be determined for the WT producing Arg-Phe due to the low product concentrations.

The best variants for Arg-Ser (T81F_A158S) and Arg-Phe production (K83F_S156A) were selected for further characterization of the activity and reusability. Since 25 and 30 °C at pH 8.5 led to the highest product concentrations with the wild-type enzyme (Figure 5b), both were tested. Time courses for product formation were determined (Figure 6a), and the reusability of the immobilisates was examined by recycling them after 24 h reaction time over a total of seven cycles (Figure 6b).

With the increase in both substrate concentration and enzyme amount, product concentrations of up to 11.2 mM Arg-Ser were reached by T81F_A158S at 25 °C, while the wild-type enzyme produced 10.3 mM under the same conditions (Figure 6a, left panel). Similar to the previous results (Figure 4a), T81F_A158S displayed a substantially higher initial activity, producing 6.5 mM Arg-Ser during the first four hours, while the wild type only reached 3.9 mM. After that, the differences decreased, and similar product concentrations were reached after 24 h. At 30 °C, final product concentrations after 24 h were lower, with the wild type producing 7.1 mM and T81F_A158S producing 7.7 mM Arg-Ser. At this temperature, no differences were visible in the time course of the product formation (Figure 6a, right panel). As expected from the previous experiments (Figure 4b), K83F_S156A generated a significantly higher Arg-Phe concentration of 11.8 mM compared to the wild type with 2.3 mM. Similar to the Arg-Ser reaction, product concentrations were decreased at 30 °C.

Both immobilisates for Arg-Ser production retained more than 50% activity at both temperatures for at least 96 h of accumulated reaction time, corresponding to four reaction cycles. At 25 °C, T81F_A158S displayed a slightly higher retention of 76% activity at 96 h compared to the 61% of the wild type. While overall product concentrations were lower at 30 °C, both immobilisates retained a higher percentage of their activity at this temperature, with 88% and 83% for wild type and variant remaining after 96 h, respectively. In contrast, K83F_S156A displayed a very sharp decline in activity at 30 °C, with only 26% remaining after 96 h. At 25 °C, it showed a similar decrease to the Arg-Ser reactions during the first 72 h, but then sharply dropped to 39% at 96 h. At the end of the seven cycles, all Arg-Ser immobilisates had similar remaining activities ranging from 32 to 37%, while K83F_S156A displayed no significant remaining activity. Apart from enzyme inactivation, there was a noticeable loss of immobilisate from cycle to cycle during washing, as evidenced by immobilisate sedimenting in the used wash buffer. The discarded immobilisate was collected, and at the end of the seven cycles, approximately half of the immobilisate was lost from the reactions as judged by comparison of the discarded with the remaining immobilisate. For future applications, a different separation technique such as the use of small chromatography columns is thus advised. The choice of the washing buffer also had a large effect on the remaining activity as washing the immobilisates with the phosphate coupling buffer reduced activities to less than 30% in the third cycle regardless of pH or temperature (Figure S1). All major results were summarized in Table 1.

Reactions at 25 °C led to higher final product concentrations, while 30 °C generally led to higher specificities. Both immobilisates of RizA variants showed improved performances in comparison to the wild type. In the case of Arg-Ser, this mainly constituted an increase in the initial activity (Figure 4a and Figure 6a), higher specificity and a slightly increased reusability and final product concentration at 25 °C (Table 1). For Arg-Phe, K83F_S156A dramatically increased the product concentration five-fold and specificity almost ten-fold (Table 1). No time course was determined for Arg-Phe production of the wild type due to very low concentrations during the early reaction time. The calculation of the remaining activity also fluctuated highly and could not be interpreted. In comparison to K83F_S156A with the Arg-Ser reactions, the reusability was significantly lower. This could be due to phenylalanine as a substrate or a decreased stability of the variant.

In comparison to the free enzymes, activities and yields of the immobilisates were reduced. For Arg-Ser, yields of up to 33% were achieved with the wild-type enzyme and up to 47% with T81F with 30 mM substrate [35]. With 50 mM substrate, up to 23 mM Arg-Ser, corresponding to a yield of 46%, were produced [13]. The disadvantage of lower activities was partially offset by the ability to easily separate the immobilized enzymes from the reaction and reuse them. Thus, the cumulative product formation of the reusable immobilisates was already higher than for the free enzymes, which can only be used once. In order to improve this further, the apparent loss of activity could be addressed by exploring other immobilization supports or coupling techniques. Since both RizA and AckA were produced with a his-tag for affinity purification, the analogous affinity immobilization would be a plausible next step [25].

Apart from the reduced activity, a decline in product formation after the first eight hours of reaction time limited the yield and was a phenomenon witnessed both for the free and immobilized RizA and AckA systems [13]. While a decrease in enzyme activity by enzyme denaturing was plausible for the free enzymes, the reusability of the immobilisates showed that this could not be the sole factor. The possible degradation of the cofactors has already been discussed above in this section. Another likely factor is accumulation and inhibition by side products of the reaction, most notably phosphate. Phosphate is produced during the reaction both through the desired hydrolysis of ATP to ADP catalyzed by the l-amino acid ligase [6], but also over time by the inevitable hydrolysis of ATP and AcP by water [42,43], thus leading to the accumulation of inorganic phosphate. Apart from direct product inhibition by phosphate [44,45], its accumulation can lead to the precipitation of magnesium phosphate [46]. Since both RizA and AckA require Mg^2+^ as a cofactor, this could be an additional factor limiting the yield. In the sophisticated design of the biocatalytic cascade for the synthesis of islatravir, the addition of sucrose and a sucrose phosphorylase provided the depletion of phosphate [33,47].

Since regeneration of the expensive ATP is an absolute necessity for industrial application of the whole class of l-amino acid ligases, establishing an efficient ATP regeneration was an important step [13]. In order to further limit the cost of enzymes and improve downstream processing, immobilization of RizA was the next step. The presented work is, to our knowledge, the first published example of an l-amino acid ligase co-immobilized with an ATP-regenerating enzyme. While the developed immobilisates are not yet fit for practical applications due to their insufficient activities and yields, the results showed that both RizA and AckA can successfully be covalently immobilized and reused for several cycles. It is likely that both activity and stability can be improved upon in future work with different immobilization techniques. Another promising option would be to adapt the created immobilisates to a continuous flow setup, in which the reagents flow through the immobilisate producing a steady flow of products [48]. While flow chemistry is an established field [49], flow biocatalysis has only begun to expand significantly in recent years [50]. Advantages include less downtime of the reactor for cleaning/refilling between batches, improved mass transfer of substrates towards the immobilisate, simplified downstream processing and reduced product inhibition through continuous removal of products [48,51]. Apart from preventing the accumulation of phosphate, a continuous setup could also provide a steady stream of cofactors circumventing their discussed stability issues. Continuous systems present a challenge for cofactor regeneration as they are mono-directional and diffusion of the cofactor to and from the regenerating enzyme is severely limited [50]. Co-immobilization of the regenerating enzyme addresses this issue and is likely a necessity for employing l-amino acid ligases in such a system. The employed agarose support is also well suited for flow applications [22,26]. We hope that this study lays the foundation for further work in this direction and the application of both this interesting enzyme class and its equally interesting products.

## 3. Materials and Methods

### 3.1. Chemicals, Reagents and Strains

Chemicals were purchased from Carl Roth (Karlsruhe, Germany) or Sigma Aldrich (Taufkirchen, Germany) if not otherwise indicated. Enzymes for molecular biology were purchased from Thermo Fisher Scientific (St. Leon-Roth, Germany). The pET28a vector was purchased from Merck KGaA (Darmstadt, Germany). The *E. coli* strains BL21 (DE3) and TOP10 were maintained in our laboratory. Oligonucleotides were synthesized by Microsynth Seqlab GmbH (Goettingen, Germany).

### 3.2. Mutagenesis

Mutagenesis was performed as previously described [35]. In short, site-specific mutations were inserted by whole-plasmid PCR with two overlapping mutagenic primers on a construct already containing the other mutation. In the cases where neighboring codons were mutated, new primers combining both mutations (Table S1) were designed with the software SnapGene version 5.1.7 (2020) from GSL Biotech, LLC (Chicago, IL, USA). The three-step protocol started with a denaturing step at 98 °C for 30 s and was then followed by 20 cycles of 98 °C for 10 s, the annealing temperature (Table S1) for 30 s, and 72 °C for 130 s. Lastly, a final extension was performed at 72 °C for 10 min, and PCR products were stored at 8 °C. In the two-step protocol (Table S1), the elongation step at the annealing temperature was omitted.

### 3.3. Production of Soluble Enzymes

RizA, its variants and AckA were recombinantly produced in *E. coli* BL21 (DE3) and purified by affinity chromatography followed by desalting through gel filtration as previously described [13,35].

### 3.4. Immobilization

Immobilization was performed using Pierce NHS-Activated Agarose from Thermo Fisher Scientific (St. Leon-Roth, Germany). Here, 20 mg NHS-agarose was used for the experiments in Section 2.1, while 33 mg NHS-agarose was used for all other experiments. The respective amount of enzyme solution in coupling buffer (50 mM phosphate, 150 mM NaCl, pH 7.2) was added to the NHS-agarose in 2 mL reaction tubes and incubated with end-over-end mixing for 1 h at room temperature. Afterwards, the immobilisates were subjected to centrifugation at 1.0× *g* for 1 min, and the supernatant was removed, which was followed by two washing steps (with intermittent centrifugation) with 800 µL desalting buffer (50 mM tricine, 100 mM NaCl, pH 8.0). Subsequently, 800 µL quenching buffer (1 M TRIS-HCl, pH 7.4) was added, followed by end-over-end mixing for 30 min. After centrifugation, the supernatant was removed, and the immobilisates were washed three times (with intermittent centrifugation) with 800 µL desalting buffer. Finally, the immobilisates were stored at 4 °C until usage. Samples of the enzyme solution added to the NHS-agarose and samples of the supernatant after the first incubation were collected to determine of the coupling efficiency. Protein concentrations were determined with Bradford solution from Sigma-Aldrich (Taufkirchen, Germany).

### 3.5. Biocatalysis

Biocatalytic reactions with the free variants were performed as previously for comparability reasons [35]. Reactions using immobilisate were performed in a reaction volume of 500 µL with the designated amino acid concentrations, equimolar amounts of acetyl phosphate, and 7.5 mM each MgSO_4_ and ATP. In the cases where RizA or AckA were not (co-)immobilized, the designated amounts of soluble enzyme were added. Reactions were performed on a Thermomixer comfort from Eppendorf SE (Hamburg, Germany) and incubated at the designated reaction temperature and 1200 rpm. Samples were taken at the designated times and inactivated for 5 min at 70 °C in a Biometra thermal cycler from Analytik Jena (Jena, Germany) and stored at −20 °C until analysis. The immobilisates were recycled by removing the old reaction solution after centrifugation, followed by two washing steps with 800 µL desalting buffer. After removing the last washing solution, the immobilisate was used again.

### 3.6. Analysis

Product and side product analysis was performed as previously described by RP-HPLC with pre-column derivatisation using *o*-phthalaldehyde and fluorescence detection of the dipeptide derivates [13,35,52]. Data were visualized with SigmaPlot 14.5 (2020) from Systat Software GmbH (Erkrath, Germany).

## 4. Conclusions

New variants of RizA were created through the combination of the best single mutations from a former study to improve the enzyme’s activity and specificity. Both the RizA wild-type enzyme and a selection of seven variants were successfully co-immobilized with AckA for ATP regeneration. Immobilisates of the two variants with the highest activities for the production of Arg-Ser (T81F_A158S) and Arg-Phe (K83F_S156A) retained more than 50% activity for at least 96 and 72 h, respectively. The variant for Arg-Phe also significantly increased product concentration and specificity by factors of 5 and nearly 10, respectively, in comparison to the wild-type enzyme.

## Figures and Tables

**Table 1:** Comparison of the biocatalytic performances of the RizA immobilisates.

Dipeptide	Variant	Temperature (°C)	c(Arg-X)_24 h_ (mM)	c(Arg-Arg)_24 h_ (mM)	Arg-X/ Arg-Arg	Yield (Arg-X)_24 h_ (%)	>50% Activity *
Arg-Ser	Wild type	25	10.3 ± 0.8	1.9 ± 0.3	5.4 ± 1.1	21	>96 h
		30	7.1 ± 0.2	1.1 ± 0.0	6.7 ± 0.3	14	>120 h
	T81F_A158S	25	11.2 ± 0.0	1.6 ± 0.1	7.2 ± 0.6	22	>120 h
		30	7.7 ± 1.9	1.0 ± 0.0	8.1 ± 0.4	15	>96 h
Arg-Phe	Wild type	25	2.3 ± 0.2	1.5 ± 0.3	1.5 ± 0.2	5	n.d.^†^
		30	1.2 ± 0.3	1.1 ± 0.0	1.1 ± 0.3	2	n.d.^†^
	K83F_S156A	25	11.8 ± 0.4	0.7 ± 0.0	16 ± 0.2	24	>72 h
		30	9.3 ± 0.9	0.4 ± 0.2	24 ± 15	19	>48 h

* Time that the immobilisates displayed more than 50% remaining activity, ^†^ Could not be accurately determined due to overall low activity.

## Data Availability

Data are contained within the article and the Supplementary Materials.

## Supplementary Materials

The following supporting information can be downloaded at: https://www.mdpi.com/article/10.3390/molecules27144352/s1, Figure S1: Reusability of the immobilisates when using coupling buffer for washing; Table S1: Primer pairs for mutagenesis.

## Sample Availability

Samples of the plasmids for the RizA variants are available from the authors.
