# Supplementary material for: Co-Immobilization of RizA Variants with Acetate Kinase for the Production of Bioactive Arginyl Dipeptides
Source: Molecules. 2022 Jul 7;27(14):4352. doi: 10.3390/molecules27144352 (PMC9321006; doi:10.3390/molecules27144352)
Supplement: Supplementary file 1 [file molecules-27-04352-s001.zip › molecules-1775195-supplementary.pdf]

Supplementary Materials

Co-Immobilization of RizA Variants with Acetate Kinase for the Production of Bioactive Arginyl Dipeptides

Sven Bordewick, Ralf G. Berger and Franziska Ersoy

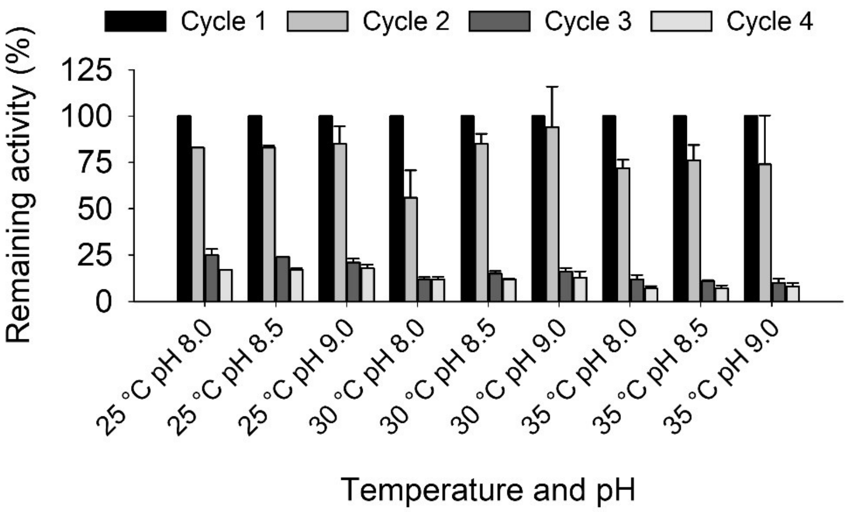

Figure S1. Reusability of the immobilisates when using coupling buffer for washing. The remaining activity was calculated as the product concentration reached after an additional cycle of 24 h in comparison to that after the first cycle. 0.4 mg of RizA and 0.04 mg AckA were used for immobilization. 50 mM of Arg and Ser were used.

Table S1. Primer pairs for mutagenesis. The given annealing temperatures are for the three-step protocol, in the two-step protocol annealing and elongation were both performed at 72°C (see Materials & Methods).

Name	Sequence (5'->3')	Annealing temperature (°C)
T81F_K83F fw	TTTGAATTTAGCATTCTGACCGGTGGTTTTC	64.4
T81F_K83F rv	AATGCTAAATTCAAAGGTGCTAACAATATG	64.4
T81F_K83R fw	TTTGAACGTAGCATTCTGACCGGTG	64.7
T81F_K83R rv	AATGCTACGTTCAAAGGTGCTAAC	64.7
T81F_S84F fw	GAAAAATTTATTCTGACCGGTGGTTTTCTGCG	65.5
T81F_S84F rv	CAGAATAAATTTTTCAAAGGTGCTAACAATATGATC	65.5
K83F_S84F fw	GAATTTTTCATTCTGACCGGTGGTTTTCTGCG	Two-step protocol
K83F_S84F rv	CAGAATGAAAAATTCGGTGGTGCTAACAATATGATC	Two-step protocol
